# The mediating effect of trauma and loss spectrum on the relationship between autistic traits and eating disorder symptoms among patients with borderline personality disorder

**DOI:** 10.1017/S1092852925100783

**Published:** 2025-12-03

**Authors:** Barbara Carpita, Chiara Bonelli, Stefano Pini, Carla Cappiello, Benedetta Nardi, Margarita Pustynnikova, Filippo Del Grande, Matteo Pioltino, Gabriele Massimetti, Ivan Mirko Cremone, Mario Luciano, Andrea Fiorillo, Lilliana Dell’Osso

**Affiliations:** 1 Department of Clinical and Experimental Medicine, University of Pisa, Pisa, Italy; 2 Department of Biotechnology, Chemistry and Pharmacy, University of Siena, Siena, Italy; 3 Department of Psychiatry, University of Campania “L. Vanvitelli”, Naples, Italy

**Keywords:** Borderline personality disorder, autism spectrum disorder, autistic traits, eating disorders, post-traumatic stress disorder

## Abstract

**Background:**

While increasing research is reporting a higher presence of autistic traits (AT) in patients with borderline personality disorder (BPD), both autism spectrum disorder (ASD) and BPD have been associated in literature with a vulnerability to trauma and eating disorders.

**Aim:**

The present study aimed at evaluating trauma-related symptoms and eating disorder symptoms in BPD patients with or without significant AT.

**Methods:**

A sample of 73 BPD patients was assessed with the adult autism subthreshold (AdAS) Spectrum, trauma and loss spectrum-self report (TALS-SR), and EDI-2 questionnaires.

**Results:**

The findings revealed that BPD patients with autistic traits (BPD-AT) scored significantly higher on the eating disorder inventory (EDI-2) and trauma and loss scale (TALS-SR) compared to those without AT. Moreover, while both AdAS Spectrum and TALS-SR scores predicted higher EDI-2 scores, a significant mediating effect of TALS-SR on the relationship between AdAS Spectrum and EDI-2 scores was reported.

**Conclusion:**

These results suggest that AT may imply more severe clinical correlates in the BPD population, including an enhanced vulnerability toward psychopathological traits frequently reported in BPD patients such as eating disorders and trauma-related symptoms, stressing the need to clarify the complex interactions among these disorders and the factors that may shape specific illness trajectories.

## Introduction

According to the Diagnostic and Statistical Manual of Mental Disorders, Text Revised (DSM-5 TR), borderline personality disorder (BPD) is a pervasive pattern of impulsivity and instability in self-image, personal relationships, and affectivity.^1^ The core symptoms include fear of being abandoned and intense efforts to avert the abandonment; paranoid ideations and dissociative symptoms during periods of intense stress, self-injuries, unmotivated and intense anger with difficulty in being able to control it.^1^^,^^2^ To date, the disorder is increasing in prevalence representing 15–20% of inpatients and causing severe consequences in patients’ lives such as emotional and behavioral dysregulation since early years, frequent school interruptions, reduced work productivity, poorer socioeconomic position.^3^^–^^5^ Moreover, BPD subjects reported high rates of suicide attempts during lifetime (79%) and suicide (10%).^3^^,^^5^ In this perspective, BPD may worsen the clinical picture of the disorders with which it commonly co-occurs: bipolar disorder, feeding and eating disorders, panic and anxiety disorders, autism spectrum disorder (ASD).^6^^–^^8^ A growing field of literature is focusing on the presence of autistic traits (AT) among patients with BPD.^2^^,^^9^ BPD and ASD share common features such as impulsive and self-harming behaviors; emotional outbursts preferred over verbalizing emotions; deficits in recognizing, distinguishing, and integrating emotions; impairment in theory of mind; deficits in social function and interaction. Moreover, some authors focused on the prevalence of subthreshold AT that may exacerbate the BPD clinical picture.^5^ In addition, ASD is considered a vulnerability factor for the development of post-traumatic stress disorder (PTSD) symptoms, increasing the vulnerability of BPD subjects toward the development of a stress-related disorder.^2^^,^^10^ Indeed, literature reported high percentage of traumatic experiences in BPD subjects, ranging from early ages to adulthood, due to problems with self‐coherence and self‐continuity, deficits in mentalizing or social cognition.^11^ Moreover, BPD shares with autism spectrum a reduced empathy and socioemotional reciprocity.^5^ In this perspective, social difficulties typical of ASD may expose subjects to repeated socially stressful events leading to the development of stress-related symptoms after milder life events ascribable to PTSD complex (cPTSD).^9^ cPTSD and BPD reported some common clinical features such as self-destructive behaviors, negative self-image, dissociation and paranoia, feelings of emptiness and numbness.^2^^,^^9^ For this reason, the two disorders may co-occur and also be frequently confused.^9^ Some authors also hypothesized that ASD females may be misdiagnosed as cPTSD/BPD due to the higher presence of these conditions in the ASD female population.^9^

During recent years, several authors stressed the fact that ASD diagnostic criteria would be tailored to the typical male presentations of the disorder, while female subjects may present specific features of this condition.^9^ Noteworthy, autistic patients reported higher rates of eating and feeding disorders (FED), while up to 50% of women with anorexia nervosa (AN) report ASD symptoms.^12^ In this perspective, the strong interest in food and diet, together with the ritualization in food preparation and consumption typical of AN, may resemble the autistic-like patterns of stereotyped interests and behaviors, explaining the opposite gender ratio between the two disorders.^9^ Moreover, AN and ASD share difficulties in social interactions and an increased risk of social isolation, difficulties in advanced theory of mind and deficits in emotional intelligence, social anhedonia, poor performance in set-shifting tests, and excellent skills in attention to detail tests, confirming the strong link between the two disorders.^13^^,^^14^ At the same time, BPD and FED frequently co-occur, especially in individuals with deficits in emotional regulation and with strong impulsivity.^15^^,^^16^ As BPD patients reported difficulties in managing intense emotional experiences and their ability to manage them is often compromised,^17^ dysfunctional food-related behaviors, such as binge eating or drastically reducing food intake, may be employed to provide temporary emotional relief.^13^^,^^14^^,^^18^ Furthermore, the BPD unstable identity and search for approval^11^^,^^19^ is also frequently reported in FEDs patients.^20^^,^^21^ The rigorous control of the body gives the impression of being able to achieve the social perfection to which these subjects aspire.^20^^,^^21^

Finally, both disorders are frequently related to a history of trauma, showing a difficulty in processing and coping with traumatic experiences^15^^,^^16^ that may contribute to the comorbidity between ASD, BPD, and FED. Indeed, AT subjects reported deficits in emotional regulation, often resulting in maladaptive coping mechanisms such as excessive or restrictive food intake.^13^^,^^22^^–^^24^

In this framework, the aim of this work was to evaluate the presence of trauma- and stress-related symptoms and eating disorder symptoms among patients with BPD with or without significant AT, focusing on investigating the specific associations among these psychopathological dimensions.

## Materials and methods

### Study sample and procedures

For the purpose of the study, we recruited a sample of 73 consecutive patients with BPD attending the Psychiatric Clinic of the University of Pisa. Participants in the study ranged in age from 18 to 65 years. Individuals under 18, those with significant intellectual or language impairments, schizophrenia, a history of substance abuse, major neurological or medical conditions, or those unable to provide written informed consent or complete psychometric questionnaires independently were excluded. Detailed information about the study was provided, and participants had the chance to ask questions before signing the written informed consent.

The study followed the ethical guidelines outlined in the Declaration of Helsinki and was approved by the ethical committee of Azienda Ospedaliera Universitaria Pisana (AOUP).

All patients were clinically assessed and fulfilled the AdAS Spectrum, trauma and loss spectrum-self report (TALS-SR), and EDI-2.

### Measures

#### The Adult Autism Subthreshold Spectrum (AdAS) spectrum

The AdAS Spectrum is a self-report questionnaire containing 160 items, designed to evaluate a broad range of autism-related traits in individuals without cognitive or language impairments. It is organized into seven domains, each focusing on specific aspects: childhood and adolescence, verbal communication, nonverbal communication, empathy, inflexibility and adherence to routine, restricted interests and rumination, and hyper- and hyporeactivity to sensory input. A validation study showed that the tool has high internal consistency, excellent test–retest reliability, and strong convergent validity with other dimensional measures of autism.^25^

#### The Eating Disorder Inventory (EDI-2)

The EDI-2 is a widely used self-report tool consisting of 91 items across 11 subscales.^26^ This version expands on the original 64 items of the EDI [B3] by adding 27 new items organized into three additional sub-scales: asceticism, impulsivity, and social insecurity. Respondents rate their answers on a 6-point Likert scale. Three of the subscales specifically address the core symptoms of eating disorders, namely drive for thinness, bulimia, and body dissatisfaction. The remaining eight subscales assess psychological traits commonly associated with eating disorders, including ineffectiveness, maturity fears, social insecurity, perfectionism, interpersonal distrust, impulsivity, interoceptive awareness, and asceticism. This broad scope allows for a comprehensive evaluation of both the behavioral and psychological aspects of eating disorders.

#### The Trauma and Loss Spectrum – Self Report (TALS-SR)

The TALS-SR is a tool designed to assess lifetime exposure to various loss and traumatic events, as well as stress-related symptoms, behaviors, and personal traits that may contribute to the risk of developing stress-related disorders. It consists of 116 dichotomous items, organized into nine domains: loss events, grief reactions, potentially traumatic events, responses to losses or distressing events, reexperiencing, avoidance and numbing, arousal, maladaptive coping, and personal characteristics/risk factors. Higher scores on the tool indicate greater symptom severity. The questionnaire has shown robust psychometric properties overall.^27^

### Statistical analysis

First, the sample was divided in two groups based on the score obtained at the AdAS Spectrum Wilson questionnaire. Specifically, subjects scoring above the threshold score of 70 were included in the BPD-AT group, while those who scored below were included in the BPD-only group. Then, student t-tests and Chi-square analysis were employed to compare age and gender in the two groups.

We then proceeded to perform further t-tests in order to compare the scored obtained by the two groups in the EDI-2 and TALS-SR domains as well as their totals.

Afterward, we used Pearson’s correlation analysis in order to investigate the presence of significant correlations between AdAS Spectrum, EDI-2, and TALS-SR total scores.

We then performed a linear regression analysis using AdAS Spectrum and TALS-SR total scores as independent variables and EDI-2 total score as dependent variable in order to evaluate whether AT or trauma- and stress-related symptoms were statistically predictive of pathological eating habits.

Given that both independent variables showed a significant predictive effect on the EDI-2 total score, a mediation analysis was then carried using the AdAS Spectrum total score as the predictor, the EDI-2 total score as the dependent variable, and the TALS-SR total score as the mediator. The Hayes PROCESS tool was utilized, and bootstrap confidence intervals for both unstandardized and standardized indirect effects were computed. All statistical analyses were performed with SPSS version 26.0.

## Results

The BPD-AT and the BPD only groups did not significantly differ in age and gender (see Table 1).

**Table 1.** Age and gender comparison between groups

		BPD only	BPD-AT	*t*	*p*
		Mean ± SD	Mean ± SD		
Age		33.31 ± 11.36	29.30 ± 11.62	1.457	.149

		*n*(%)	*n*(%)	Chi-square	*p*
Gender	F	27(93.1%)	37(84.1%)	1.314	.252
	M	2 (6.9%)	7(15.9%)		

Results from the student t-test highlighted that BPD-AT subjects scored significantly higher in EDI-2 *Interoceptive Awareness, Maturity Fears*, and *Impulsivity* domains as well as in its total. Similarly, BPD-AT group scored higher on TALS-SR *Reaction to Losses or Upsetting Events*, *Maladaptive Coping, Arousal* and *Personal Characteristics and Risk Factors* domains as well as in its total (see Table 2).

**Table 2.** Comparison of EDI-2 and TALS-SR scores between the two groups

	BPD only	BPD-ASD	*t*	*p*
	mean ± SD	mean ± SD		
EDI–2				
Drive for thinness	7.45 ± 7.51	8.18 ± 6.79	−.433	.667
Bulimia	3.14 ± 3.79	4.64 ± 5.21	−1.420	.160
Body dissatisfaction	8.86 ± 6.73	8.64 ± 5.43	.158	.875
Ineffectiveness	7.59 ± 4.75	9.89 ± 5.57	−1.826	.072
Perfectionism	4.79 ± 3.72	6.73 ± 4.35	−1.966	.053
Interpersonal distrust	4.90 ± 3.55	6.09 ± 4.48	−1.206	.232
Interoceptive awareness	7.07 ± 5.65	10.45 ± 7.06	−2.179	.033*
Maturity fears	5.52 ± 4.35	8.00 ± 5.46	−2.153	.035*
Asceticism	5.31 ± 3.07	6.89 ± 4.08	−1.775	.080
Impulsivity	5.69 ± 3.97	10.48 ± 7.99	−3.388	.001*
Social insecurity	5.59 ± 5.43	7.57 ± 4.96	−1.608	.112
Total score	65.90 ± 31.39	87.54 ± 41.20	−2.541	.013*
TALS-SR				
Loss events	4.34 ± 1.74	4.78 ± 1.74	−1.010	.316
Grief reactions	13.41 ± 5.89	14.28 ± 6.44	−.578	.565
Potential Traumatic Events	6.41 ± 3.49	6.58 ± 3.26	−.208	.836
Reaction to losses or upsetting events	8.41 ± 4.25	10.56 ± 3.93	−2.196	.031*
Reexperiencing	4.17 ± 2.71	5.35 ± 2.50	−1.894	.062
Avoid. and numb.	4.83 ± 3.11	6.02 ± 2.61	−1.764	.082
Maladaptive coping	2.24 ± 2.06	3.60 ± 2.31	−2.651	.013*
Arousal	2.10 ± 1.65	3.23 ± 1.57	−2.927	.005*
Pers. charact./risk factors	2.38 ± 1.29	3.19 ± 1.72	−2.146	.035*
Total score	48.31 ± 20.52	57.58 ± 16.63	−2.110	.038*

*Note:* *significant for *p* < .005.

Results from Pearson’s correlation analysis showed significant positive correlations between AdAS Spectrum, EDI-2, and TALS-SR total scores in the overall sample (see Table 3).

**Table 3.** Pearson’s correlation coefficients between AdAS spectrum, EDI-2, and TALS-SR total scores in the overall sample

	AdAS spectrum total score	EDI-2 total score
AdAS spectrum total score		.437*
TALS-SR total score	.374*	.373*

*Note:* *significant for *p* < .005.

Results from the linear regression analysis, performed using AdAS Spectrum and TALS-SR total scores as independent variables and EDI-2 total score as dependent variable, showed how both questionnaires were significant positive predictors of higher EDI-2 scores (see Table 4).

**Table 4.** Linear regression performed using AdAS Spectrum and TALS-SR total scores as independent variables and EDI-2 total score as dependent variable

	B(SE)	Beta	*t*	*p*
*Constant*	9.210(15.388)		.599	.551
AdAS spectrum total score	.537(.176)	.345	3.052	.003*
TALS-SR total score	.510(.236)	.244	2.161	.034*

*Note: R*^2^:.242; Adjusted *R*^2^: .220; *significant for *p* < .005.

**Figure 1.**
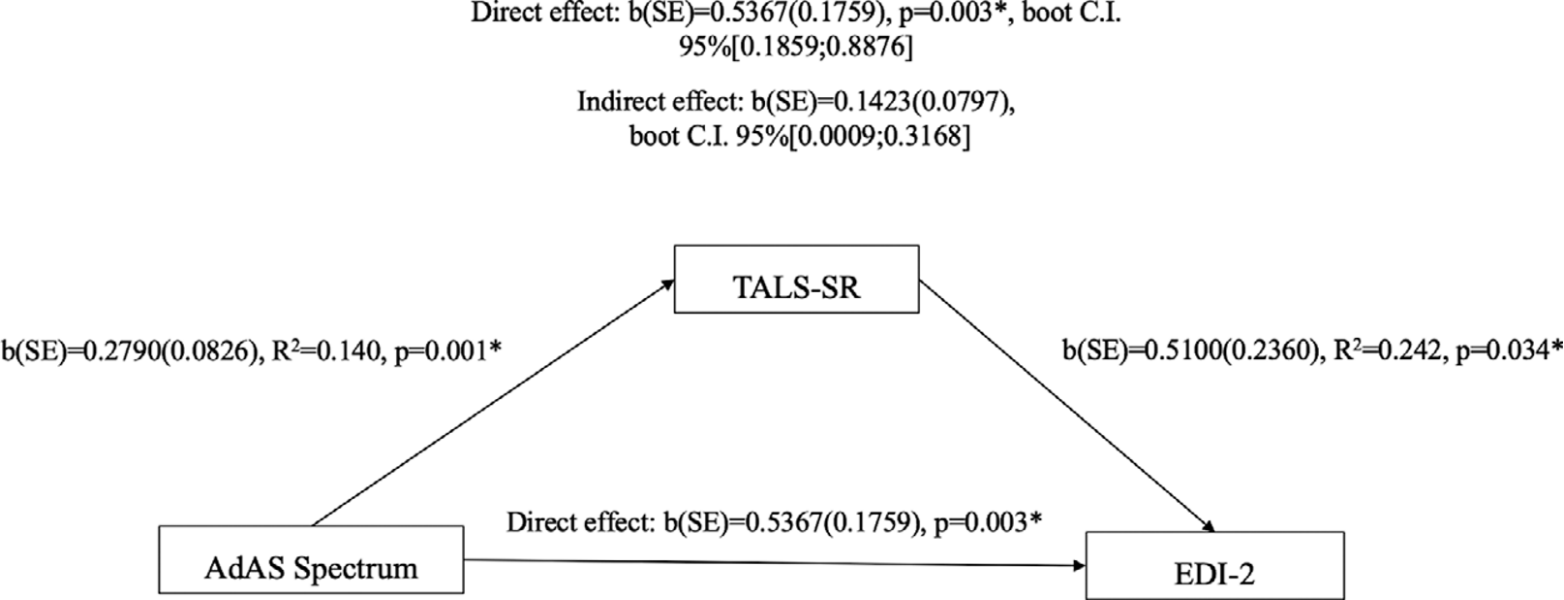
Mediation analysis results.

The results from the mediation analysis demonstrated significant direct effect of 0.5367 (*p* = .003) of the AdAS Spectrum total score on the EDI-2 total score. Moreover, the AdAS Spectrum total score also exhibited a significant indirect effect on the EDI-2 total score through the TALS-SR total score (*b* = 0.01423, 95% bootstrapped confidence interval (CI) [0.0009:0.3168]) (see Figure 1).

## Discussion

The aim of this work was to evaluate the presence of trauma- and stress-related symptoms and eating disorder symptoms among patients with BPD with or without significant AT, focusing on investigating the specific associations among these psychopathological dimensions.

Comparing the two groups, BPD-AT one scored significantly higher on the EDI-2 and TALS-SR total scores compared to BPD subjects. These results are in line with the currently available literature, which demonstrates the presence of a greater vulnerability to the development of eating disorders in individuals with AT, as well as a higher presence of AT in FED patients, who share with ASD subjects several features, such as restricted interests, inflexibility, and social difficulties.^14^ Interesting findings come from a recent meta-analysis exploring potential associations between AT and eating disorder symptoms. The authors selected 22 studies highlighting a significant increase in AT of patients with Feeding and Eating Disorder (FED) than Healthy Controls (HC). Moreover, 29% of FED subjects scored above the cut-off for ASD diagnosis at the Autism Diagnostic Observation Schedule (ADOS) questionnaire.^28^ On the basis of these findings, some authors even hypothesized that AN could be reconceptualized as a female phenotype of the autism spectrum.^12^^–^^14^ On the other hand, the presence of an autistic trait facilitates a lifetime exposure to various traumatic events as well as a greater vulnerability to develop stress-related symptoms after the traumatic experience.^29^ Our results confirm the existence of an association between autism spectrum and trauma and stress symptoms in BPD subjects, supporting the hypothesis already present in the literature that individuals in the autism spectrum share with BPD a greater vulnerability to traumatic events, and that, eventually, this vulnerability may be at least partially underlain by AT in this population.^30^^–^^32^ AT frequently expose and are more vulnerable to traumatic events, also due to a deficit in socio-emotional reciprocity^33^^–^^35^ and a high tendency to rumination, leading to the development of post-traumatic symptoms and full-blown PTSD.^34^^,^^36^ At the same time, BPD subjects diagnosed with PTSD would seem to have a higher autism spectrum burden.^2^

Moreover, BPD-AT subjects scored significantly higher in some specific domains. The higher scores reported on the *Impulsivity* domain (EDI-2) compared to the BPD group may be in line with the lack of inhibitory control in ASD, which may eventually facilitate impulsive and, eventually, compulsive behaviors related to eating habits.^5^^,^^14^^,^^37^^,^^38^

Our results also reported that BPD-AT subjects reported higher scores on EDI-2 *Interoception* domain, evaluating the ability to be aware of internal sensations in the body, as well as emotional sensations.^39^ While alexithymia and altered interoceptive awareness are reported in BPD,^40^ as well as in FED,^41^ to the point that this domain was included in a questionnaire assessing FED-related symptomatology, they are also typical features of ASD.^41^ Indeed, in autistic subjects, the deficit in detecting and attending to internal bodily sensations moderating the experience of body ownership may affect not only social function and empathic abilities but also emotional experiences often linked to maladaptive eating behaviors.^42^ Eventually, it could be hypothesized that altered interoceptive awareness and alexithymia would be underlain by AT in these populations.^5^^,^^43^ In addition, the BPD-AT group scored higher also on the EDI-2 *Maturity Fears* domain. While according to the literature, subjects with FED may show increased fear and uncertainties related to growth and transition to adult life, including body changes^44^; it is possible that the inflexibility and adherence to routine typical of subjects with ASD, with an impaired ability to adjust to changes, may also favor fear and denial of transition to adulthood.^45^

As expected, the BPD-AT group also reported interesting results in specific TALS-SR domains. The presence of a significant score at the *Arousal* domain confirmed previous data about the presence in ASD of altered arousal regulation.^46^^–^^48^ The reduced arousal threshold leading to brain abnormal response to the external environment may cause an intrinsic vulnerability to stressful triggers’ processing,^47^^–^^49^ explaining, therefore, the higher total score of the BPD-AT group at the questionnaire. Noteworthy, the difficulty in managing inputs coming from the external world is often compensated by maladaptive behaviors, in line with the higher scores reported in the *Maladaptive Coping* domain to reduce the persistence and intensity of traumatic experience in autistic subjects.^49^ Moreover, BPD-AT subjects reported higher scores on TALS-SR *Reaction to losses or upsetting events* and *Personality characteristics/risk factors.* This finding is in line with the reported difficulties underlying by AT toward mentalizing loss or traumatic events and subsequently adjusting to them due to the tendency toward ruminative thinking and inflexibility traits in these patients.^49^ On the other hand, AT may be at the basis of specific personality traits which are considered as risk factors for the development of trauma- and stress-related symptoms, such as those related to impulsivity and emotional dysregulation, which are reported in ASD patients, and higher sensibility to stress.^5^^,^^50^^,^^51^ The intertwined relationship between AT, FED, and trauma- or stress-related symptoms in these patients is further supported by the significant correlations reported in our findings. While as described above, the link between AT and both trauma- or stress-related symptoms and FED has been widely reported in the literature, on the other hand, FED and a history of trauma-related conditions may often present in comorbidity.^52^

Findings from the linear regression analysis indicate that both AdAS Spectrum and TALS-SR total scores significantly predict higher EDI-2 scores, suggesting a strong association of eating disorder symptomatology with both autism spectrum traits and trauma-related symptoms. While trauma-related symptoms, such as reexperiencing and maladaptive coping, have been studied widely in people with eating disorders,^52^ ASD symptoms, including subthreshold manifestations, are gaining increasing attention as a potential risk factor for developing such disorders, specifically in the context of female phenotypes of autism spectrum. Indeed, individuals with elevated AdAS Spectrum scores may exhibit rigid thinking, difficulty with emotional regulation, and sensory processing issues, which are commonly implicated in eating disorders.^53^ In particular, according to the raising hypothesis which frames AN as a possible female phenotype of an autism spectrum, the specific interest in food and diet, as well as the repetitive behaviors involving eating may constitute a specific presentation of autistic-like restrictive interests and repetitive behaviors in FED patients. On the other hand, FED patients often present social difficulties, social anhedonia, and an impaired theory of mind, with a profile of this latter similar to those with ASD^14^ This connection is further reinforced by research suggesting both a higher prevalence of FED in patients with ASD and a higher prevalence of AT in FED, particularly AN.^12^^,^^14^

Finally, AdAS Spectrum total score exhibited a direct effect on EDI-2 total score, as well as a significant indirect effect on the EDI-2 total score through the TALS-SR total score, highlighting the complex interplay between autism spectrum traits, trauma exposure, and eating disorder pathology. This relationship suggests that trauma-related symptoms may act as a mediator between autism spectrum traits and disordered eating behaviors. Such a relationship could be explained by the fact that individuals with autism spectrum traits often have heightened sensitivity to environmental and interpersonal stressors, which may amplify the psychological impact of traumatic experiences.^18^ In turn, traumatic experiences and post-traumatic symptoms, which are common in BPD patients,^52^ have been consistently associated with increased vulnerability to eating disorders, as individuals may engage in maladaptive eating behaviors, such as restriction or binge eating, as a coping mechanism.^22^ Globally, this finding may support the hypothesis that, in BPD patients, AT may act as a vulnerability factor toward the development of eating disorders directly, in line with the association between AT and FED reported in the literature, but also indirectly, toward enhancing the vulnerability toward the development of trauma- and stress-related symptoms, which, in turn, may facilitate the development of eating disorders. This model would be in line with the above-reported associations between AT and FED, between AT and trauma- and stress-related symptoms, and, finally, between trauma and stress-related symptoms and FED, offering an integrated model for explaining the intertwined interactions among these conditions.

This study should be considered in light of some limitations. The first limitation could be represented by the use of self-report instruments, which may facilitate bias related to over- or underestimation of symptoms by participants. Moreover, the small sample size and the statistical analyses investigated only sex and age as sociodemographic factors, even though the homogeneity in the groups limits the confounding factors related to these demographic features. Finally, our cross-sectional study is based on a purely statistical predictivity not allowing us to make cause–effect inferences or temporal relationships.

Globally, our work confirmed the presence of higher eating disorder symptoms and trauma- and stress-related symptoms in BPD patients with AT, suggesting that AT may imply more severe clinical correlates in this population, including an enhanced vulnerability toward psychopathological traits frequently reported in BPD patients such as eating disorders and trauma- and stress-related symptomatology. Moreover, a specific mediating role of trauma- and stress-related symptoms in mediating the relationship between AT and eating disorder was highlighted. These findings may shed light on the interaction between AT and post-traumatic symptoms, both conditions associated with BPD, in shaping the vulnerability toward eating disorders in these patients.

## Data Availability

All data generated or analyzed during this study are included in this published article.
